# Real-time structure search and structure classification for AlphaFold protein models

**DOI:** 10.1038/s42003-022-03261-8

**Published:** 2022-04-05

**Authors:** Tunde Aderinwale, Vijay Bharadwaj, Charles Christoffer, Genki Terashi, Zicong Zhang, Rashidedin Jahandideh, Yuki Kagaya, Daisuke Kihara

**Affiliations:** 1 Department of Computer Science, Purdue University, West Lafayette, IN 47907 USA; 2 Department of Biological Sciences, Purdue University, West Lafayette, IN 47907 USA

**Keywords:** Protein structure predictions, Molecular modelling

## Abstract

Last year saw a breakthrough in protein structure prediction, where the AlphaFold2 method showed a substantial improvement in the modeling accuracy. Following the software release of AlphaFold2, predicted structures by AlphaFold2 for proteins in 21 species were made publicly available via the AlphaFold Database. Here, to facilitate structural analysis and application of AlphaFold2 models, we provide the infrastructure, 3D-AF-Surfer, which allows real-time structure-based search for the AlphaFold2 models. In 3D-AF-Surfer, structures are represented with 3D Zernike descriptors (3DZD), which is a rotationally invariant, mathematical representation of 3D shapes. We developed a neural network that takes 3DZDs of proteins as input and retrieves proteins of the same fold more accurately than direct comparison of 3DZDs. Using 3D-AF-Surfer, we report structure classifications of AlphaFold2 models and discuss the correlation between confidence levels of AlphaFold2 models and intrinsic disordered regions.

## Abstract (web summary)

3D-AF-Surfer is presented as a computational resource for real-time protein structure comparison search between AlphaFold2 models and PDB entries within seconds to a few minutes.

## Introduction

Structural biology has entered a phase when structure prediction methods, particularly a recent method, AlphaFold2^1^, consistently produce reliable computational structure models with atomic accuracy. Protein structure prediction has been extensively studied in the computational biology community. Taking advantage of the accumulated protein sequence and structure information in the Protein Data Bank (PDB)^2^, numerous methods have been developed based on different scientific disciplines, ideas, and various computational techniques. In the past few years, methods that use machine learning methods, particularly deep neural networks^3–9^, made a large improvement in structure prediction accuracy in the Critical Assessment of techniques in protein Structure Prediction (CASP)^10^. In CASP14, a breakthrough^11^ was achieved by AlphaFold2^1^, which showed the best performance among participants with a substantial gap to the second-best method. Remarkably, the accuracy of AlphaFold2 models often reaches what would be expected from X-ray crystallography. It has been reported that models generated by AlphaFold2 have indeed helped experimental protein structure determination, as such models were successfully used for molecular replacement in X-ray crystallography and for density interpretation of cryo-EM maps^12,13^.

Soon after the release of the AlphaFold2 code, predicted structure models by AlphaFold2 for proteins from 21 major model species have been released at the AlphaFold Protein Structure Database^14^. This is an invaluable resource for the biology community as modeled protein structures can be easily obtained without installing and running the AlphaFold2 software. Many proteins that do not have experimentally determined structures now have computational models with an expected high accuracy.

Here, we provide the infrastructure, 3D-AF-Surfer, for real-time protein structure model search within AlphaFold2 models and across entries in PDB at https://kiharalab.org/3d-surfer/submitalphafold.php. In any database, the functionality for quick entry search and comparison is essential. In 3D-AF-Surfer, a quick structure search against the entire PDB and AlphaFold2 models is realized with 3D Zernike descriptors (3DZD), which are rotationally invariant, mathematical representations of 3D shapes^15,16^ (see Methods for more technical details). 3DZDs were shown to be effective in rapid protein structure database search^17–20^ other tasks that involve biomolecular shape comparison and matching^21–25^, mapping the global shape space of known protein structures^26^, binding pocket comparison^27,28^, drug screening^28,29^, and protein docking^22^. To the best of our knowledge, 3D-AF-Surfer is the only tool that can search between AlphaFold2 models and PDB entries real-time, within seconds to a couple of minutes. In 3D-AF-Surfer, we further developed neural networks that take 3DZDs of proteins as input and achieve more accurate retrieval of proteins of the same fold than a direct comparison of 3DZDs.

## Results

### Domains with high confidence in AlphaFold2 models

In 3D-AF-Surfer, protein structure models generated by AlphaFold2 for 21 proteomes were retrieved from the European Bioinformatics Institute’s FTP server of the AlphaFold Database (https://ftp.ebi.ac.uk/pub/databases/alphafold) on July 22, 2021, which is still up-to-date on November 8, 2021. AlphaFold2 assigns one of four confidence levels, from very high confidence to very low confidence, to each amino acid position in a model. The confidence levels were assigned by the predicted local distance difference test (pLDDT) score^30^, which examines the accuracy of Cα atom distances in a model. Since many models have low or very low confidence regions, which often have unfolded conformation, we extracted confident domain region(s) from each model in 3D-AF-Surfer (see Methods). In total, this procedure yielded 508,787 domains, which cover 48.8% of residues in all the AlphaFold2 models. The statistics of model counts is provided in Table 1.

**Table 1:** Proteomes and structure models considered.

Species	Common name	Reference proteome	# unique UniProt IDs	# original	# domains	# structure predictions with no domains (1D)
*Arabidopsis thaliana*	Arabidopsis	UP000006548	27,434	27,434	37,682	5722
*Caenorhabditis elegans*	Nematode worm	UP000001940	19,694	19,694	26,160	4277
*Candida albicans*	*C. albicans*	UP000000559	5974	5,974	9,978	743
*Danio rerio*	Zebrafish	UP000000437	24,664	24,664	42,135	2530
*Dictyostelium discoideum*	*Dictyostelium*	UP000002195	12,622	12,622	18,963	2986
*Drosophila melanogaster*	Fruit fly	UP000000803	13,458	13,458	19,881	2335
*Escherichia coli*	*E. coli*	UP000000625	4363	4363	5397	417
*Glycine max*	Soybean	UP000008827	55,799	55,799	72,217	14,146
*Homo sapiens*	Human	UP000005640	20,504	23,391	44,827	3302
*Leishmania infantum*	*L. infantum*	UP000008153	7924	7924	12,257	1579
*Methanocaldococcus jannaschii*	*M. jannaschii*	UP000000805	1,773	1,773	2,097	131
*Mus musculus*	Mouse	UP000000589	21,615	21,615	35,216	2477
*Mycobacterium tuberculosis*	*M. tuberculosis*	UP000001584	3988	3988	5170	351
*Oryza sativa*	Asian rice	UP000059680	43,649	43,649	39,775	19,756
*Plasmodium falciparum*	*P. falciparum*	UP000001450	5187	5187	7283	1162
*Rattus norvegicus*	Rat	UP000002494	21,272	21,272	33,818	2664
*Saccharomyces cerevisiae*	Budding yeast	UP000002311	6040	6040	9837	967
*Schizosaccharomyces pombe*	Fission yeast	UP000002485	5128	5128	8173	637
*Staphylococcus aureus*	*S. aureus*	UP000008816	2888	2888	3283	415
*Trypanosoma cruzi*	*T. cruzi*	UP000002296	19,036	19,036	26,205	5436
*Zea mays*	Maize	UP000007305	39,299	39,299	48,433	11,582

For each proteome, the number of unique proteins, total original/domain models, and total original models containing no confident domains are given. The definition of the confident domains is given in the main text. The human original model count is underlined, indicating that the number of original models does not match the number of unique proteins. The human structure predictions retrieved from the AlphaFold Database contain models which are 1400-residue slices of larger proteins.

### 3D-AF-Surfer

**Fig. 1:**
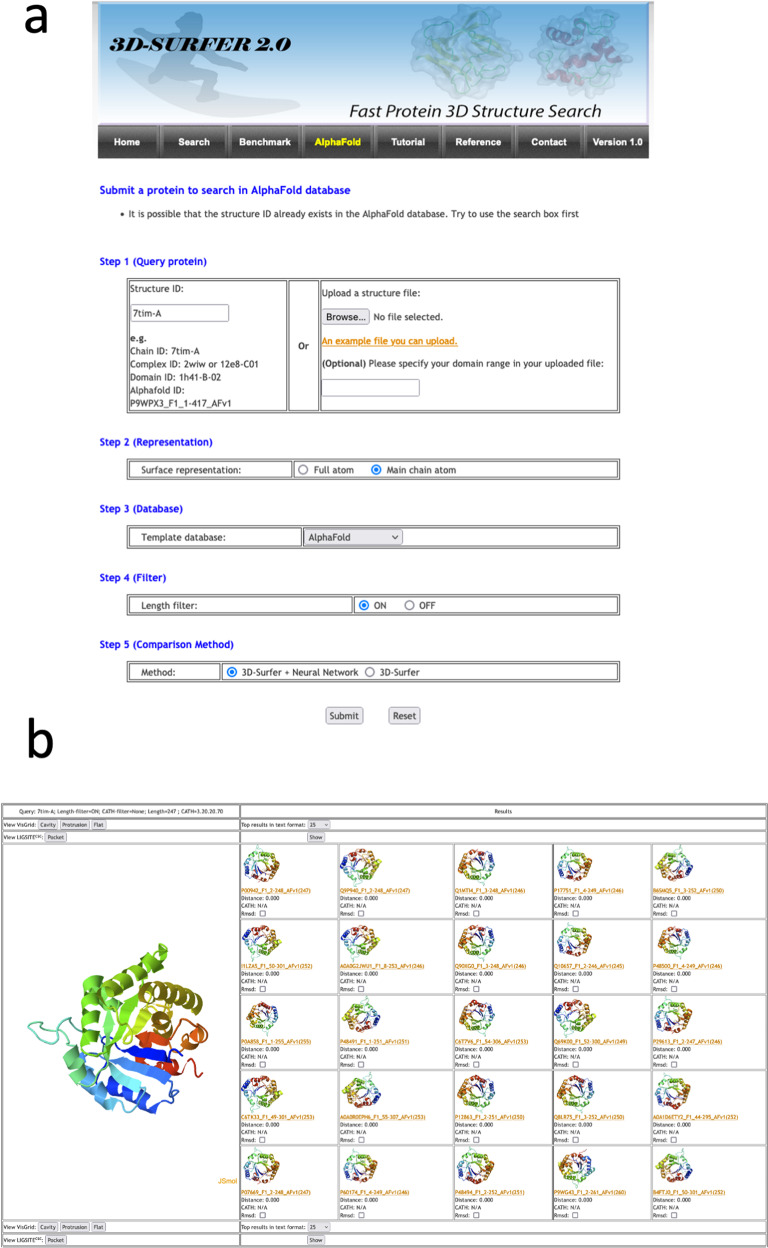
Input and an output example of 3D-AF-Surfer. **a** The input page (see text). **b** An example output page. The query was PDB ID: 7tim-A, a TIM-barrel fold and search was against AlphaFold2 models using the deep neural network. As shown, retrieved top 25 hits are all TIM-barrel folds with a distance of 0.0, indicating that the network judged that these structures are highly likely to belong to the same fold.

Figure 1 illustrates the input and output panels of 3D-AF-Surfer, available at https://kiharalab.org/3d-surfer/submitalphafold.php. In the input panel, users can enter the AlphaFold model ID, PDB ID or upload the file of the query structure (Fig. 1a). When the first couple of letters of ID is entered, candidates of the rest will be listed. Then, the representation of protein structures used to compute 3DZD needs to be specified (full atom or main chain). Next, select the database to search against, which can be the full AlphaFold proteome database, structures from PDB (complexes, domain structures) or both combined. Users also have an option to select the method of the database search, a deep neural network-based search (the default setting), which is suitable for retrieving proteins with the same fold (see below) or original 3DZD-based search that is equipped in 3D-Surfer. The result page shows a table where the query structure is displayed on the left side and a list of retrieved structures ranked by their similarity to the query is shown on the right side (Fig. 1b). Clicking a retrieved structure invokes a new search using the selected structure as the query and allow users to “surf” in the protein structure universe. The panel also provides the option to compute the root mean square deviation (RMSD) between the query and the displayed similar structure. Pockets in the query structure can be identified using VisGrid^31^ or LIGSITE^32^. Finally, shown at the bottom of the page is the 3DZD of the query structure.

PDB entries in 3D-AF-Surfer are updated bi-weekly. As of November 29, 2021, the server holds 547,639 protein chains and 249,163 additional domain structures from PDB, and 508,787 domain structures from the AlphaFold Database. Average time for a search measured over ten queries is as follows, when the neural network is used: Against AlphaFold domains: 55 s (s); PDB chains: 1 min 10 s; PDB domains: 22 s; PDB chains+domains: 1 min 15 s; All of the above: 2 min 26 s. Search is faster if 3DZD is used: 3 s against AlphaFold domains; 1.35 s, 1.45 s, 1.93 s against PDB chains, domains, and chains+domains, respectively, and 2.45 s for All of the above.

We further compared the computational time of 3D-AF-Surfer with DaliLite^33^, TM-align^34^, MADOKA^35^, SPalignNS^36^, and ZEAL^37^. DaliLite and TM-align are conventional, commonly used structure alignment methods, while MADOKA and SPalignNS are more recent methods. ZEAL is a method that uses 3D Zernike moments instead of 3DZDs (see “Methods”). Table 2 reports the computational time of these methods on structure comparison of 4950 protein pairs formed from randomly sampled 100 proteins. For 3D-AF-Surfer, both direct 3DZD comparison and the neural network (3DZD-NN) were evaluated. 3DZD is the fastest of all methods, followed by 3DZD-NN. MADOKA was the next fastest, but it was 10 times slower than 3DZD-NN. ZEAL was the slowest of all the methods.

**Table 2:** Comparison of computational time.

Method	Running time
3DZD	1.64 s
3DZD-NN	4.06 s
DaliLite	4 min 37.2 s
TM-align	10 min 14.4 s
MADOKA	41.4 s
SPalignNS	19 min 18.55 s
ZEAL	3 days 3 h 22 min 7.47 s

We ran the programs on a Linux machine with an Intel(R) Core i7-6900K CPU @ 3.20 GHz. min, minutes; sec, seconds. The running times reported are the average of three independent runs.

### Secondary structure class of AlphaFold2 models

**Fig. 2:**
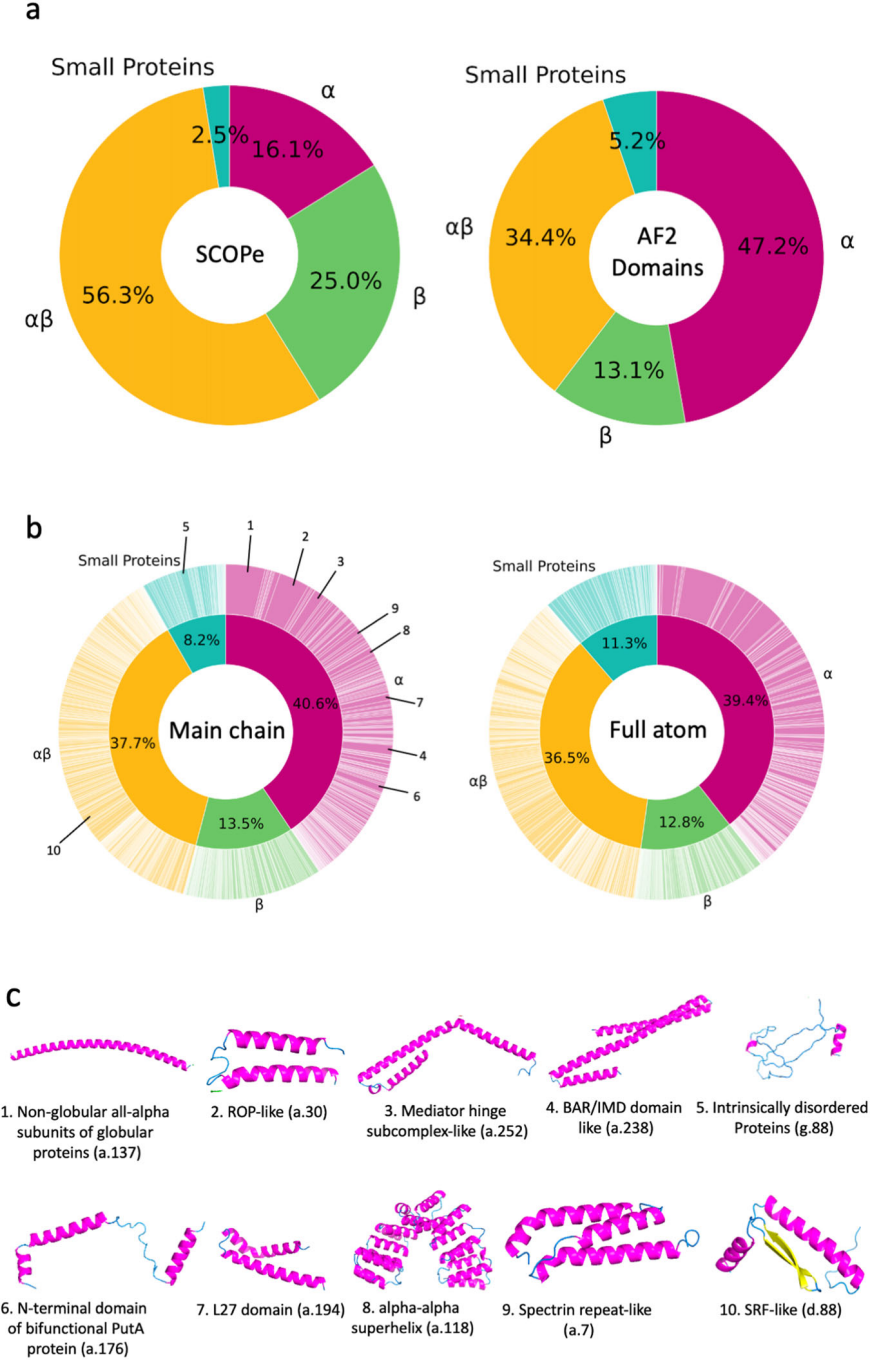
Distribution of protein secondary structure classes and fold classes of confident domains of AlphaFold2 models. **a** The secondary structure classes were assigned to SCOPe domains and domains of high confidence in AlphaFold2 models. Four classes were considered, α, β, αβ, and small proteins. Left, SCOPe (232,630 domains); right, domains of high confidence in AlphaFold2 models. (508,787 domains). The classification was performed using a bagged SVM ensemble (see Methods). SCOPe domains (left) were also classified with the SVM ensemble to be able to compare with the results on AlphaFold2 domains (right). **b** Fold classification of the AlphaFold2 structure domains of high confidence. The classification was performed with the deep neural networks that were trained on the fold assignment provided in SCOPe (see Methods). The outer wheel indicates the fraction of each fold. Folds were ordered according to SCOPe IDs. Left, the fold distribution of AlphaFold2 domains using the deep network trained on 3DZDs of full atom domain structure surface. The inner wheel shows the fraction of secondary structure classes. Since this classification was based on the fold assignment, the fractions are overall consistent but not identical to those shown in panel (**a**). The top 10 most abundant folds are indicated. Right, the fold distribution using the deep network trained on 3DZDs of surface shapes with main-chain atoms. **c** The 10 most abundant folds among AlphaFold2 domains. The fraction of each fold is indicated in the wheel diagram on the left in panel b. For each fold, an example of AlphaFold domains is shown. (1) Non-globular all-alpha subunits of globular proteins (a.137). Example shown is A0A1D6E4Z3_F1, residue 823-895 (*maize*). (2) ROP-like (a.30): A0A1D6MV33_F1, residue 758-815 (*maize*). (3) Mediator hinge subcomplex-like (a.252). Q4DL50_F1, residue 384-495 (*T. cruzi*). (4) BAR/IMD domain-like (a.238). Q8LE58_F1, residue 2-133 (*Arabidopsis*). (5) Intrinsically disordered proteins (g.88). I1L2C2_F1, residue 210-284 (*soybean*). (6) N-terminal domain of bifunctional PutA protein (a.176). A7MBM2_F1, residue 157-225 (*human*). (7) L27 domain (a.194). A0A1D6PKM6_F1, residue 314-375 (*maize*). (8) alpha-alpha superhelix (a.118). K7KHY8_F, residue 213-524 (*soybean*). (9) Spectrin repeat-like (a.7). P38637_F1, residue 149-238_AFv1 (*S. cerevisiae*). 10 SRF-like (d.88). A0A1D6NUQ9_F1, residue 2-74 (*maize*).

Figure 2a shows a breakdown of the secondary structure class of domain structures of AlphaFold2 models in comparison with SCOPe^38,39^. Four secondary structure classes were considered, α, β, αβ, and small proteins. αβ corresponds to the α+β and α/β classes in SCOPe. The classification was performed with a machine learning method, a bagged^40^ ensemble of support vector machine classifiers (SVMs) using the secondary structure content of SCOPe domains (see Methods). The bagged ensemble had an accuracy of 91.5% (Table 3). The method had the highest accuracy among all the methods compared, which include handmade classification procedures and different architectures of SVM. The classification result for SCOPe (Fig. 2a) is qualitatively consistent with earlier statistics of CATH^41^, where the αβ class occupies over 50% and the share of α-class is around 15%. On the other hand, we note a greater prevalence of α-class structures among the AlphaFold2 domains (Fig. 2b) than in the SCOPe statistics (Fig. 2a). This result probably indicates that α-class structure models tend to have higher confidence than other classes.

**Table 3:** Accuracy of fold class assignment on SCOPe.

Method	Accuracy				
	Overall	$\alpha$	$\beta$	$\alpha \beta$	Small proteins
Expert handmade (without optimization)	0.852	0.683	0.771	0.961	0.357
Expert handmade (optimized)	0.880	0.759	0.889	0.928	0.500
Multinomial logistic regression	0.863	0.916	0.861	0.851	0.818
SVM (linear)	0.445	0.991	0.927	0.069	0.548
SVM (RBF kernel)	0.896	0.947	0.869	0.896	0.861
Bagged SVM (RBF kernel)	0.915	0.943	0.882	0.937	0.621

Fold classes were assigned to AlphaFold2 models based on secondary structure content and sequence length. Here we show the benchmark results from optimizing these classifiers on the original manually curated SCOPe fold classes. For the expert handmade classifiers, secondary structure content and protein length conditions were defined for each fold class. The first classifier without optimization used the following conditions: ${{{{{{\rm{length}}}}}}} < 50{aa}\to {{{{{{\rm{small}}}}}}}$; else ${{{{{{\rm{helix}}}}}}}\ge 60 \% \to \alpha$; else ${{{{{{\rm{sheet}}}}}}}\ge 35 \%$ and ${{{{{{\rm{helix}}}}}}} < 20 \% \to \beta$; else $\to \alpha \beta$. The second one optimized the actual threshold values by parameter sweep of an increment of 5% for secondary structure content and increments of 5aa for the sequence length. The optimized mapping was: ${{{{{{\rm{length}}}}}}} < 55{aa}\to {{{{{{\rm{small}}}}}}}$; else ${{{{{{\rm{helix}}}}}}}\ge 55 \% \to \alpha$; else ${{{{{{\rm{sheet}}}}}}}\ge 25 \%$ and ${{{{{{\rm{helix}}}}}}} < 20 \% \to \beta$; else $\to \alpha \beta$. For the other classifiers, lengths and secondary structure proportions were used directly as features. For each classifier, accuracy is shown both overall and per-class.

### Fold classification by deep neural network

**Fig. 3:**
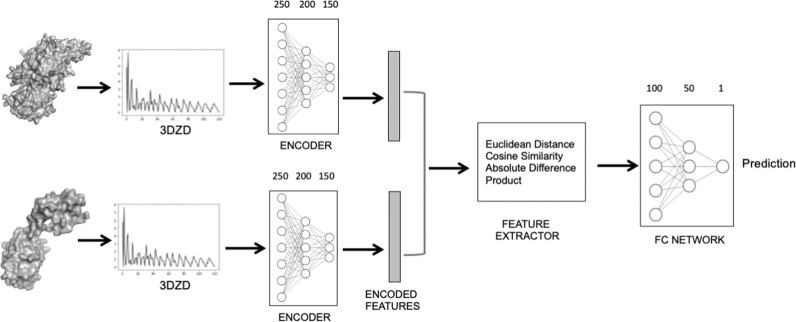
Deep neural network model for protein fold classification. The Network takes as input two protein structures represented by their 3DZD vectors. The encoder layer uses the three hidden layers, each with 250, 200, 150 nodes, to encode the features in the 3DZD. The encoding vector of a length of 1452 is then input into the feature extractor layer, which is used to compare the encoded feature of the two structures using four distance metrics, the Euclidian distance, the cosine distance, the Manhattan (absolute value) distance, and dot product. The FC network takes the feature extractor output and predicts the probability that the two structure belong to the same fold.

To have an overall grasp of the fold distribution of AlphaFold2 models, we used the deep neural network of 3D-AF-Surfer and classified AlphaFold domain structures into SCOPe folds (Fig. 2b). For this classification, we considered 1101 folds in the class a (all α proteins), b (all β proteins), c (α/β proteins), d (α+β proteins), and g (small proteins) in the SCOPe database. The neural network takes 3DZDs of two protein structures and outputs the probability that the two structures belong to the same SCOPe fold^42^ (Fig. 3; see “Methods”). This neural network architecture has shown significant performance in the yearly-held 3D Shape Retrieval Contests (SHREC) protein retrieval categories^42,43^.

For the current work we newly trained two networks, one that uses 3DZDs computed from full-atom protein surface and the other one that takes 3DZDs computed from main-chain Cα, C, and N atoms^44^. The network with the main-chain atoms showed higher classification accuracy (95.0%) than the full-atom network (Table 3). This accuracy was higher than the original 3D-Surfer^17^, which compares 3DZDs directly with the Euclidean distance.

We also compared the structural classification performance of 3DZD and 3DZD-NN with SPalignNS, because Janan et al.^45^ performed a comprehensive analysis of eighteen structure alignment methods and reported SPalignNS as the best method for fold classification (Supplementary Fig. 1). This comparison was performed on randomly sampled 2,500 positive (i.e. same-fold) and 2,500 negative (i.e. different-fold) pairs from the validation dataset used in Table 4. As shown in the figure, 3DZD-NN showed the highest AUC of 0.998, followed by SPalignNS with an AUC of 0.976. The AUC of 3DZD was the lowest, at 0.789.

**Table 4:** Fold classification accuracy by 3DZD and the deep neural network.

Method	3DZD Type	Accuracy	Precision	Recall	F-Measure
Fold					
3DZD-NN	Full Atom	0.954	0.945	0.964	0.954
	Main Chain	0.977	0.974	0.979	0.977
3DZD	Full Atom	0.508	0.504	0.998	0.670
	Main Chain	0.616	0.571	0.939	0.710

This benchmark is computed using the test set from the SCOPe dataset. Balanced positive and negative test pairs were constructed from the set of 2521 protein structures in SCOPe. There were 167,872 test pairs in total. 3DZD is the original method where the 3DZD of two structures are compared with a score that uses Euclidean distance of 3DZDs of two proteins, which is defined as 1/(1+Euclidean distance). Thus, the score ranges from 0 to 1. 3DZD-NN is the deep network that outputs predicted probability that input two structures are in the same SCOPe fold. Probability values output by 3DZD-NN range from 0 to 1. We used the best threshold that maximized F-measure. The threshold values of 3DZD-NN full atom, 3DZD-NN main-chain, and 3DZD were 0.5, 0.6, and 0.1, respectively. See Table 1 in Supplementary Information for results of all different thresholds. See Methods for definitions of accuracy, precision, recall, and F-measure.

### Illustrative cases of misclassifications of folds

**Fig. 4:**
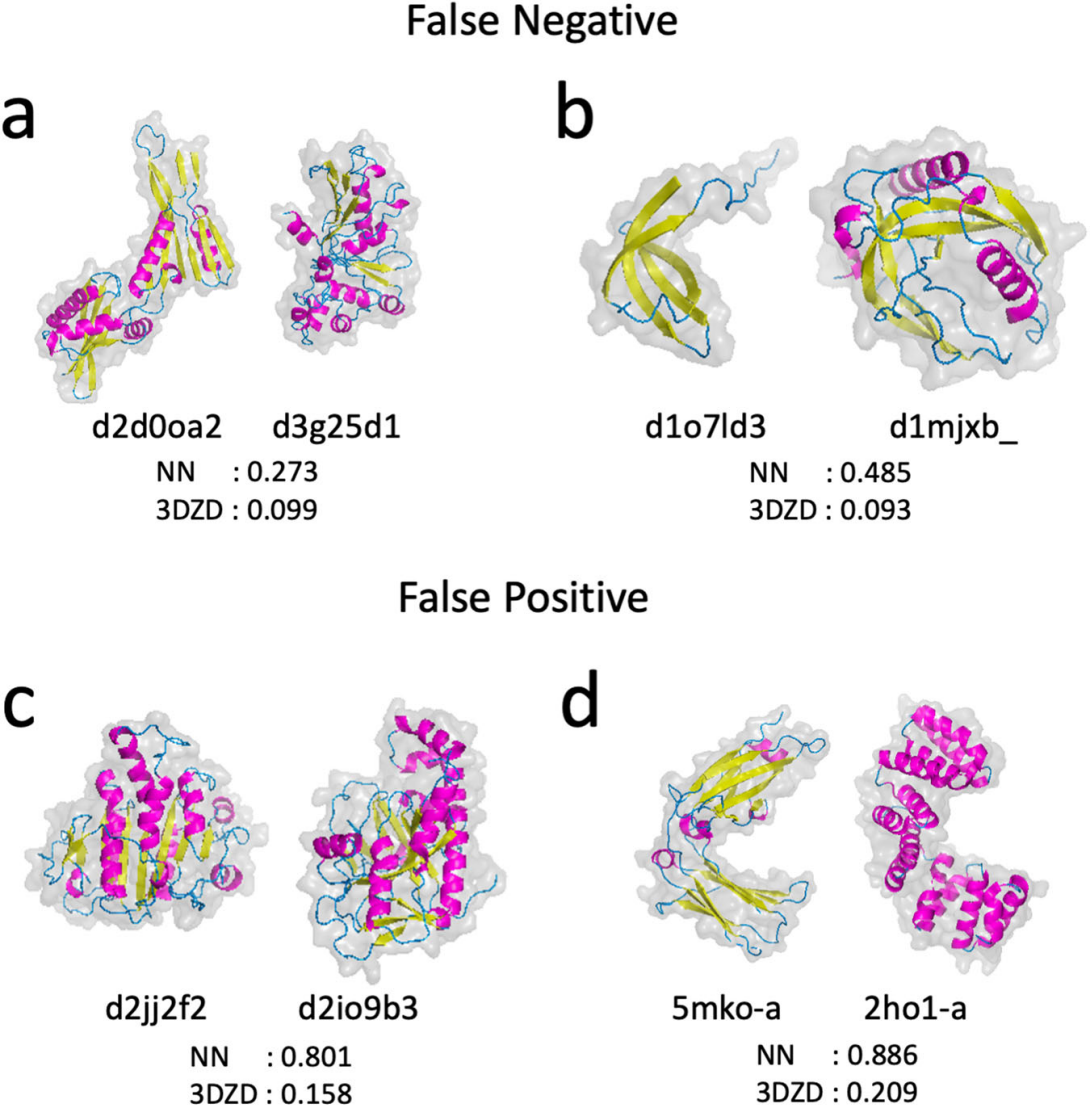
Examples of protein pairs that were misclassified by 3D-AF-Surfer. Four protein structure pairs are shown with scores from 3DZD-NN and 3DZD. 3DZDs of the main-chain atoms were used. The two numbers below each protein pair are scores of the two structures by 3DZD-NN and 3DZD. The pairs on panel a and b are cases where both 3DZD-NN and 3DZD considered the two proteins to belong to different SCOP folds but they actually do not. **a** d2d0oa2 and d3g25d1 belong to the Ribonuclease H-like motif fold (SCOP code: c.55). The scores of 3DZD-NN and 3DZD for this pair was 0.273 and 0.099, respectively, both of which were lower than threshold values used (0.6 and 0.1) and thus considered as different folds. **b** d1o7ld3 and d1mjxb_ belong to OB fold (b.40). The two pairs on panel c and d are examples of false positives, where 3D-AF-Surfer suggested that each pair belonged to the same fold, but they actually do not. **c** d2jj2f2 belongs to P-loop containing nucleoside triphosphate hydrolases (c.37) while d2io9b3 belongs to ATP-grasp (d.142). **d** PDB ID: 5mko-A is a β class structure while 2ho1-A is an α-class structure. These two structures do not have a SCOP ID assigned at time of writing. This example is taken from the paper that reported ZEAL^37^.

Although 3D-AF-Surfer showed high fold classification performance as discussed above, there are certainly cases where it failed to provide a correct classification. Some such cases come from the inherent methodology of using 3DZDs as discussed in our earlier paper^19^. We showed four examples in Fig. 4. The two pairs in panel a and b are false negatives where the two structures belong to the same SCOP fold while both 3DZD-NN and 3DZD considered them as different folds. The pair in Fig. 4a (d2d0oa2 and d3g25d1) have similar secondary structure arrangement along the sequences but their spatial packings are different. Consequently, these two structures have different overall surface shape for 3DZD. In the pair in Fig. 4b, although the two structures have a bent β-sheet structure in common, extra α-helices in d1mjxb_ made the two folds less similar, which also led to differences in their surface shapes.

Fig. 4c, d shows examples of false positives, i.e. two pairs of structures of different folds where both 3DZD-NN and 3DZD recognized them as the same fold. The structures in Fig. 4c have similar spatial arrangements of secondary structures, each with a large β-sheet in the middle and a long, kinked characteristic α-helix on the side, although structure superimposition shows an RMSD over 15 Å. Figure 4d shows two proteins with different secondary structure classes but with a similar C-shaped surface shape. Detecting similar surface shape of proteins regardless of their main-chain conformations is characteristic of the performance of 3DZD, which, in these two cases, led to false positives. However, note that while these false positive pairs have a score above the detection threshold, they do not practically affect a database search against the entire PDB or AlphaFold2 models because there are many far more similar structures that occupy top hits in a search as shown in Supplementary Fig. 2.

**Fig. 5:**
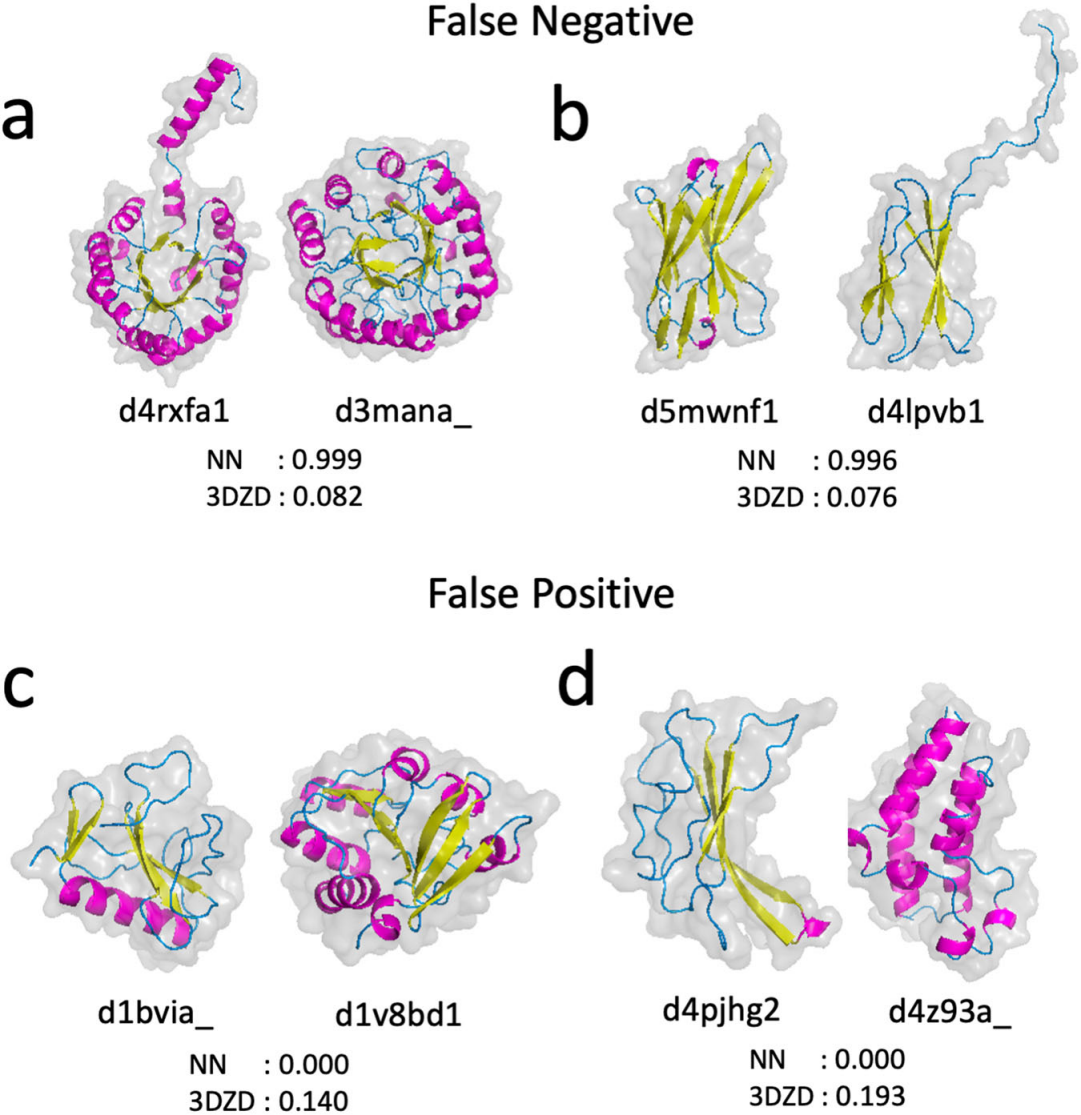
Examples of pairs where 3DZD-NN classified correctly but 3DZD did not. Four protein structure pairs with the scores by 3DZD-NN and 3DZD are shown. The main-chain atom representation was used for these comparisons. The two pairs on panel a and b are cases where 3DZD did not recognize them as the same fold (i.e. false negative) but 3DZD-NN did. **a** d4rxfa1 and d3mana_ belong to the TIM beta/alpha-barrel (SCOP code: c.1). 3DZD-NN had a probability score of 0.99, i.e. very confident that these two structures belong to the same fold while 3DZD had a score of 0.082, below the detection threshold of 0.1. **b** d5mwnf1 and d4lpvb1, belong to Immunoglobulin-like beta-sandwich (b.1). Panel c and d shows two pairs, where 3DZD-NN correctly detected that the two structures have different folds while 3DZD had a score above the threshold of 0.1 and thus considered them as the same fold. Fold assignment of the four structures in SCOP are as follows: **c**, d1bvia_: Microbial ribonucleases (d.1); d1v8bd1: NAD(P)-binding Rossmann-fold domains (c.2); (**d**), d4pjhg2: Immunoglobulin-like beta-sandwich (b.1); d4z93a_: Bromodomain-like (a.29).

In Fig. 5 we discuss cases where 3DZD-NN improved over 3DZD, where the neural network correctly classified two proteins as being in the same fold or not while 3DZD failed. In the pairs in Fig. 5a, b surface shapes of the two proteins are apparently different due to a tail that flipped out from the main body of the protein volume. 3DZD was confused by the shape difference, but the neural network was still able to correctly identify the pair as belonging to the same fold with high confidence. Figure 5c and Fig. 5d show cases where 3DZD had a slightly higher score than the threshold and considered them as the same fold while 3DZD-NN considered them as different folds. In both cases, while 3DZD could not differentiate the pairs due to their similar surface shapes, the neural network is able to differentiate the pair as not belonging to the same fold.

To summarize, surface shape similarity of proteins, which 3DZD detects, can lead to misclassification of protein folds if that is the main interest of users. But in many cases the neural network was able to correct such misclassification by 3DZD. It would be worthwhile to note that identifying proteins with similar surface shape but different main-chain conformations by 3DZD often lead to findings of functionally related proteins, which were otherwise missed due to the lack of main-chain and sequence-level similarity^19,37^.

### Fold distribution of AlphaFold2 models

We now discuss abundant folds observed in Alphafold2 models. In Fig. 2b, the fold classification are shown in wheel diagrams. The inner and the outer wheels of the pie charts show the classification result at the secondary structure class level and at the individual SCOPe folds, respectively. The distribution of the secondary structure class levels is consistent with Fig. 2a, which was classified from secondary structure content of models. Classifications using the main-chain atoms (the left panel in Fig. 2b) and full-atoms (the right panel) were also consistent. Overall, the α-class folds are dominant when all the proteomes are considered.

In Fig. 2c, we showed 10 most abundant folds from all the 21 species. Among them, eight belong to the α-class, one to the α+β-class (d.88), and one to the small protein class (g.88), respectively. Supplementary Table 2 breaks down the statistics into individual species. Reflecting the overall abundance of α-class proteins as shown in Fig. 2, α-class folds dominate top 10 rankings in all the species. On average, 7.0 α-class folds ranked within top 10 in each species, which contrasts to the small numbers of folds in α/β or α+β-class (1.67 folds) and β−class (0.71 folds). These results of Alphafold2 models are largely different from statistics taken from the SUPERFAMILY2.0 database^46^, which is a reference of the current understanding of protein fold distribution (Supplementary Table 3, 4). As shown in Supplementary Table 4, the 21 species in SUPERFAMILY2.0 have more α/β or α+β-class folds within top 10: On average, 5.24 folds from the α/β or α+β-class are within top 10, which contrasts with 1.9 α-class folds. The dominance of the α/β and α+β-class observed in SUPERFAMILY2.0 is consistent with earlier works by Gerstein^47^, which is shown in Supplementary Table 5 and by Kihara & Skolnick^48^ (Supplementary Table 6), which assigned folds by a threading method. In Supplementary Table 2, commonly appeared folds with the SUPERFAMILY2.0 statistics (Supplementary Table 4) are underlined. There are not many common folds between the two tables. Seven species did not have common folds. For the rest of species, there were one to three common folds.

### Low-confidence regions of AlphaFold2 models

**Fig. 6:**
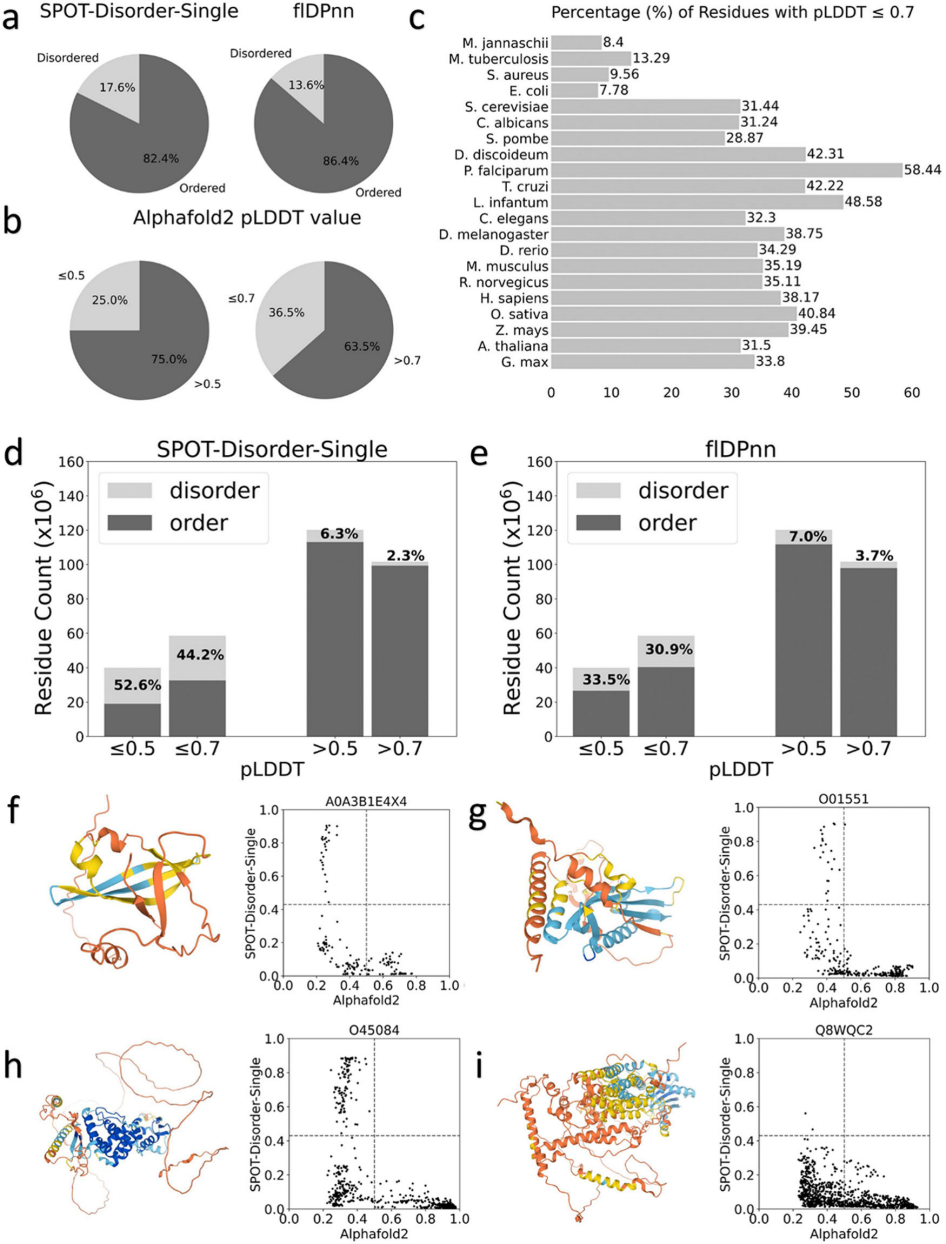
Correlation between predicted disordered regions and low-confidence regions in AlphaFold2 models. **a** Percentages of residues that were predicted as disordered or ordered by SPOT-Disorder-Single (left) and flDPnn (right). **b** Percentages of residues that were with a low confidence score $\le$ 0.5 (left) and $\le$ 0.7 (right). **c** percentages of residues with a low confidence score $\le$ 0.7 for each proteome. **d** The number of residues in predicted disordered regions in low-confidence regions with 0.5, 0.7 cutoff. prediction was made by SPOT-Disorder-Single**. e** The same type of analysis as panel d using disorder region prediction by flDPnn**. f**–**i** Case studies of correlation between the confidence score and disorder propensities by SPOT-Disorder-Single. The AlphaFold2 model ID is provided at the top of the plot. Left, the model structure. The color code shows the confidence level as used in the AlphaFold Database: blue (pLDDT > 90), light blue (90 > pLDDT>70), yellow (70 > pLDDT>50), orange (pLDDT<50). Right, correlation between the confidence score (*x*-axis) and disorder propensity (*y*-axis) for each residue by SPOT-1D-Single.

At last, we also analyzed low-confidence regions of AlphaFold2 models as they are not handled in 3D-AF-Surfer and thus left out from the above analysis. Particularly, we analyzed correlation between the low-confidence regions (pLDDT $\le$ 0.5 and 0.7) from AlphaFold2 models and disorder predictions. We used two disorder prediction methods, SPOT-Disorder-Single^49^ and flDPnn^50^. According to the two methods, about 14–18% of residues are disordered (Fig. 6a). On the other hand, considering 0.5 and 0.7 pLDDT as cutoffs, more residues, 25% and 36.5%, in AlphaFold2 models were in low confidence regions (Fig. 6b). The percentage of low-confidence residues varies for different species. Low-confidence regions are relatively small (7–13%) in the four bacterial proteomes, while *D. discoideum* has the largest fraction of low-confidence residues, 58.4%. For the other species, low-confident residues share about 30–40%.

In Fig. 6d, e, we compared disorder predictions and the model confidence scores using two score cutoffs, pLDDT of 0.5 and 0.7. When SPOT-Disorder-Single was used for disorder prediction (Figs. 6d), 52.6% and 44.2% of low-confidence regions defined with a pLDDT cutoff of 0.5 and 0.7, respectively, were predicted as disordered. Thus, reversely, 47.4% and 55.8% of low-confidence regions were predicted as ordered. On the other hand, almost all high confident regions were predicted to be ordered. The result was essentially the same when flDPnn was used (Fig. 6e), except that disordered residues in low-confidence regions became even less, 33.5% and 30.9% using pLDDT of 0.5 and 0.7 as a cutoff, respectively. The results indicate that low-confidence regions do not always correspond to disordered regions, at most only 30 to 50%, and rest would be folded in native protein structures. Figure 6f–i shows several examples. The first three panels (f, g, h) are similar cases. Low-confidence residues at pLDDT around 0.4 or lower have a wide range of disorder propensities, and about half of such residues have low disorder propensity and probably would be folded in the native structures. In the model shown in Fig. 6i does not have residues with high disorder propensity, implying that the protein would be well folded in the native form.

## Discussion

We developed 3D-AF-Surfer, which performs protein structure comparison against the entire PDB and the entire Alphafold2 models within a couple of minutes. Thus, it would be a BLAST^51^ sequence database search tool-equivalent for 3D protein structure database search. At the time of writing, there is no other method that can perform such a fast structure comparison for the entire Alphafold2 models and PDB. As demonstrated in Results, 3D-AF-Surfer maintains high accuracy yet is still able to perform a real-time structure search, which allows users to analyze Alphafold2 models interactively. Currently, 3D-AF-Surfer is running on a single CPU on a regular Linux machine and all searches are performed on the fly. Therefore, further speed up can be easily achieved by using multiple CPUs or by applying other standard techniques of database management. With such an expansion of the server, 3D-AF-Surfer will be able to handle the future release of more structure models by the Alphafold database, which is expected to happen in near future.

## Methods

### Extraction of confident domain regions in AlphaFold2 models

To extract a confident domain in an AlphaFold2 model, we first extracted all contiguous regions of more than 50 confident residues that have a pLDDT score greater than 70.0. Then, confident regions separated by at most 5 non-confident residues were merged, along with the intervening residues regardless of confidence level. AlphaFold2 models were discarded if they have no confident domains. In total, this procedure yielded 508,787 domains. 83,615 (22.9%) models out of 365,198 total AlphaFold2 models contain no confident domains. The statistics of model counts is provided in Table 1. In terms of total residues, the domain dataset in 3D-AF-Surfer contains 48.8% (78,133,986 residues) of residues among the residues in all the AlphaFold2 models (160,235,650 residues).

### SCOPe benchmark dataset for structure classification

We downloaded the latest version of the SCOPe dataset release 2.07 from the download page of the SCOPe website (https://scop.berkeley.edu/downloads/). The dataset included 256,391 structures in 1,430 folds after removing structures in class I (Artifacts). For each of the protein structures we used EDTSurf^52^ to generate the solvent excluded surface, for which a 3DZD vector is computed. We computed two types of 3DZD vector for a structure. The first one is computed using full atom of the protein structure. The second 3DZD is computed using only the main-chain Cα, C, and N atoms from the structure, because this main-chain surface representation performed better in our previous work^44^.

### Classification of secondary structure class with bagged SVM

The fold classification was performed with a bagged ensemble of SVMs using the secondary structure content of SCOPe domains. In bagging, $N$ = 20 different classifiers were trained on 5% of the SCOPe dataset selected randomly with replacement. The output classes were then decided by voting. On the training set, the bagged ensemble had an accuracy of 91.5%. This accuracy was higher than five other methods we compared, which were a multinomial logistic regression, two SVM architectures, and two expert-designed approaches. In the expert-designed approaches, the secondary structure content thresholds, i.e. fraction of amino acids in a protein in α helices, β strands, and coil (other structures) were considered. A detailed comparison of these methods is provided in Table 2.

### Performance metrics

We measured the performance of the method using Accuracy, Precision, Recall and F-measure.

1 $${{{{{\rm{Accuracy}}}}}}=\frac{{{{{{{\rm{TP}}}}}}}+{{{{{{\rm{TN}}}}}}}}{{{{{{{\rm{TP}}}}}}}+{{{{{{\rm{TN}}}}}}}+{{{{{{\rm{FP}}}}}}}+{{{{{{\rm{FN}}}}}}}}$$

2 $${{{{{\rm{Precision}}}}}}=\frac{{{{{{{\rm{TP}}}}}}}}{{{{{{{\rm{TP}}}}}}}+{{{{{{\rm{FP}}}}}}}}$$

3 $${{{{{\rm{Recall}}}}}}=\frac{{{{{{{\rm{TP}}}}}}}}{{{{{{{\rm{TP}}}}}}}+{{{{{{\rm{FN}}}}}}}}$$

4 $${{{{{\rm{F}}}}}}-{{{{{{{\rm{Measure}}}}}}}}=2{{\cdot }}\frac{{{{{{{\rm{Precision}}}}}}}\,{{\cdot }}\,{{{{{{\rm{Recall}}}}}}}}{{{{{{{\rm{Precision}}}}}}}\,+\,{{{{{{\rm{Recall}}}}}}}}$$

where TP = True positive, FP = False positive, TN = True negative, FN = False negative. True positive is the case where the protein pairs belong to the same fold and the method predicts correctly that they are in the same fold. True negative is similar to TP, the case where the protein pairs belong to different folds and the method predicts correctly that they belong to different folds.

False positive is the case where the protein pairs belong to different fold and the method predicts wrongly that they are in the same fold. False negative is the case where the protein pairs belong to the same fold and the method predicts wrongly that they belong to different folds.

### 3D Zernike descriptors (3DZD)

3DZDs are mathematical rotation-invariant moment-based descriptors. For a protein structure, a surface from a set of atoms was constructed and then mapped to a 3D cubic grid of size N3 (*N* = 200). Each voxel (a cube defined by the grid) is assigned either 1 or 0; 1 for a surface voxel that locates closer than 1.7 grid intervals to any triangle defining the protein surface, and 0 otherwise. This grid was considered as a 3D function $f\left(x\right)$, for which a series was computed in terms of the Zernike–Canterakis basis^15^:

5 $${Z}_{{nl}}^{m}\left(r,\vartheta ,\varphi \right)={R}_{{nl}}\left(r\right){Y}_{l}^{m}\left(\vartheta ,\varphi \right)$$

with $-l < m < l,0\le l\le n,$ and $(n-l)$ even. ${Y}_{l}^{m}\left(\vartheta ,\varphi \right)$ are spherical harmonics. ${R}_{{nl}}\left(r\right)$ are radial functions defined by Canterakis, constructed so that ${Z}_{{nl}}^{m}\left(r,\vartheta ,\varphi \right)$ are homogeneous polynomials when written in terms of Cartesian coordinates. 3D Zernike moments of $f\left(x\right)$ are defined as the coefficients of the expansion in this orthonormal basis, i.e. by the formula

6 $${\Omega }_{{nl}}^{m}=\frac{3}{4\pi }\int _{\left|x\right|\le 1}f\left(x\right)\,{\bar{Z}}_{{nl}}^{m}(x){{{{{{\rm{d}}}}}}x}$$

3D Zernike moments will change if the 3D object, *f(****x****)*, is rotated to a different orientation. Thus, they could be used to evaluate differences of shapes convolved with differences in orientation of two objects or to align objects^37^. To achieve rotation invariance, the moments are collected into (2 *l*+1)-dimensional vectors ${\Omega }_{{nl}}=({\Omega }_{{nl}}^{l},{\Omega }_{{nl}}^{l-1},{\Omega }_{{nl}}^{l-2},{\Omega }_{{nl}}^{l-3},\ldots {\Omega }_{{nl}}^{-l})$, and the rotationally invariant 3D Zernike descriptors ${F}_{{nl}}$ are defined as norms of the vectors ${\Omega }_{{nl}}$^21^. Thus,

7 $${F}_{{nl}}=\,\sqrt{\mathop{\sum }\limits_{m=-l}^{m=l}{\left({\Omega }_{{nl}}^{m}\right)}^{2}}$$

Index *n* is called the order of the descriptor. The rotational invariance of 3D Zernike descriptors means e.g. that calculating ${F}_{{nl}}$ for a protein and its rotated version would yield the same result. We used 20 as the order because it gave reasonable results in our previous works on protein 3D shape comparison^17,19,44,53^. A 3DZD with an order *n* of 20 represents a 3D structure as a vector of 121 invariants^19^.

### Deep neural network for fold classification

Using the generated 3DZD, we trained a deep neural network that outputs the probability that a given pair of protein structures belong to the same fold. The network (Fig. 3) takes the 3DZDs of two protein shapes as input. Three hidden layers have 250, 200, and 150 neurons, respectively, which were used as the encoding of an input 3DZD. The encoder is connected to the feature extractor, a fully-connected network, which takes the 3DZDs of the two proteins, and the encodings from the three hidden layers, and four metrics that compare two vectors, the Euclidian distance, the cosine distance, the element-wise absolute difference, and the element-wise product, and the two features of the two protein shapes (the difference in the number of vertices and faces). In total, the number of the input features of the feature comparator is 2*121 + 2 * (250 + 200 + 150) + 2 * 4 + 2 = 1,452 features. The first term is the 3DZDs of order 20 (*n* = 20), which is a 121-element vector of the two protein shapes. The third term, 2 * 4 comes from the four-comparison metrics applied to two representations of the two proteins, the original 3DZDs and encodings, which concatenate the output of the input layer and the three intermediate layers of the encoder. The feature comparator outputs a score between 0 and 1 using a sigmoid activation function, which is the probability that the two proteins are in the same fold classification in the SCOPe database.

The training and validation were performed on the aforementioned structure dataset of SCOPe. Out of 256,391 structures in 1430 unique folds, we set aside 2541 structures for model validation. For each of the structures in the database, we generated positive and negative pairs. Positive pairs are protein structures that belong to the same fold, while negative pairs are from different folds. For training, we randomly sampled a balanced set of positive and negative pairs based on the batch size (i.e. 32 positive pairs and 32 negative pairs for a batch size of 64). We used ADAM for parameter optimization with a binary cross-entropy loss function. The learning rate was explored from 1e−3 to 7e−3 and 0.1–0.7 in our previous work and set to 0.005^42^. The accuracy of networks was evaluated on the negative and positive set generated from the 2541 structures, which totals 167,872 pairs.

To assign a fold to a query protein, the query was compared with 10 randomly selected structures from each SCOPe fold. Then, the fold that showed the highest probability for the query is assigned. Although the training of each network was performed on the folds for all the classes except for the artifact class (class I), in the pie charts in Fig. 2 we assigned to folds that belong to α, β, αβ (α+β and αβ), and small proteins, because the other classes are consider factors other than structural features.

### Disorder region prediction methods

We used two methods, flDPnn^50^ and SPOT-Disorder-Single^49^. flDPnn uses profile information computed by three other methods, which is processed by a deep learning architecture to output residue-wise disorder prediction. flDPnn showed the top performance in the most recent Critical Assessment of protein Intrinsic Disorder prediction (CAID) experiment^54^. Following the instruction of the software, residues with a disorder propensity score above 0.3 were considered disordered. We used the open-sourced implementation and trained models at http://biomine.cs.vcu.edu/servers/flDPnn/.

SPOT-Disorder-Single is a fast method that computes prediction from the single sequence of the query. It uses an ensemble of nine models. At their core, each model is constructed from ResNet blocks and/or LSTM BRNN blocks. Following the instruction of the software, residues with a disorder propensity score above 0.426 were considered disordered. We adopted the local version of SPOT-Disorder-Single available at (http://sparks-lab.org/server/SPOT-Disorder-Single) and kept the default configuration.

### Statistics and reproducibility

The computational run time experiments (Table 2) were performed three times. We reported the parameters used to reproduce SCOPe database fold classification and released the trained neural network to reproduce the AlphaFold2 database fold classification.

### Reporting summary

Further information on research design is available in the Nature Research Reporting Summary linked to this article.

## Supplementary information

Supplementary Information

Reporting Summary

## Data Availability

Data used in this webserver were obtained from PDB and the AlphaFold Database and are fully and freely available to public.

## Code availability

The webserver described in this work is freely available for public at https://kiharalab.org/3d-surfer/submitalphafold.php. The codes used for classifying a protein structure into secondary structure class and fold are made available at https://github.com/kiharalab/3d-af_surfer.
