# Supplementary material for: Real-time structure search and structure classification for AlphaFold protein models
Source: Commun Biol. 2022 Apr 5;5:316. doi: 10.1038/s42003-022-03261-8 (PMC8983703; doi:10.1038/s42003-022-03261-8)
Supplement: Supplementary file 1 — Supplementary Information [file 42003_2022_3261_MOESM1_ESM.pdf]

Supplementary Information for

Real-Time Structure Search and Structure Classification for AlphaFold Protein Models

Tunde Aderinwale[1,†], Vijay Bharadwaj[1,†], Charles Christoffer[1], Genki Terashi[2], Zicong Zhang[1], Rashidedin Jahandideh[1], Yuki Kagaya[2] & Daisuke Kihara[1,2,*]

[1] Department of Computer Science, Purdue University, West Lafayette, Indiana, 47907, USA

[2] Department of Biological Sciences, Purdue University, West Lafayette, Indiana, 47907, USA

† These authors contributed equally.

* Corresponding author: E-mail: dkihara@purdue.edu

Supplementary Table 1. Fold classification accuracy by 3DZD and the deep neural network.

Method	3DZD Type	Threshold	Accuracy	Precision	Recall	F-Measure
3DZD-NN	Full Atom	0.1	0.903	0.839	0.997	0.911
		0.2	0.929	0.881	0.993	0.933
		0.3	0.943	0.908	0.987	0.945
		0.4	0.951	0.928	0.977	0.952
		**0.5**	**0.954**	**0.945**	**0.964**	**0.954**
		0.6	0.953	0.959	0.945	0.952
		0.7	0.946	0.971	0.919	0.944
		0.8	0.930	0.983	0.875	0.926
		0.9	0.879	0.994	0.763	0.863
	Main Chain	0.1	0.928	0.877	0.996	0.933
		0.2	0.95	0.913	0.994	0.952
		0.3	0.962	0.936	0.991	0.963
		0.4	0.969	0.951	0.988	0.969
		0.5	0.974	0.964	0.984	0.974
		**0.6**	**0.977**	**0.974**	**0.979**	**0.977**
		0.7	0.976	0.982	0.97	0.976
		0.8	0.969	0.989	0.948	0.968
		0.9	0.941	0.993	0.888	0.938
3DZD	Full Atom	0.05	0.500	0.500	1.000	0.667
		**0.1**	**0.508**	**0.504**	**0.998**	**0.670**
		0.15	0.624	0.604	0.717	0.656
		0.2	0.622	0.823	0.312	0.452
		0.3	0.523	0.999	0.047	0.089
		0.4	0.507	1.000	0.014	0.028
		0.5	0.503	1.000	0.005	0.010
		0.6	0.501	1.000	0.002	0.004
		0.7	0.500	1.000	0.001	0.002
		0.8	0.500	1.000	0.000	0.000
		0.9	0.500	1.000	0.000	0.000
	Main Chain	0.05	0.500	0.500	1. 000	0.667
		**0.1**	**0.616**	**0.571**	**0.939**	**0.710**
		0.15	0.679	0.739	0.553	0.632
		0.2	0.608	0.966	0.223	0.363
		0.3	0.527	0.999	0.055	0.104
		0.4	0.511	1.000	0.022	0.043
		0.5	0.505	1.000	0.010	0.019
		0.6	0.502	1.000	0.004	0.008
		0.7	0.500	1.000	0.001	0.002
		0.8	0.500	1.000	0.000	0.000
		0.9	0.500	1.000	0.000	0.000

Fold classification accuracy using different score cutoff values. The classifications were computed on a dataset of 167,872 protein pairs constructed from 2,521 protein domain structures from SCOPe. Positive and negative pairs were balanced. First, we made all possible protein pairs from the same fold, which turned out to be 83,936. Then we downsampled negative pairs to match the number of the positive pairs. Protein pairs were input to 3DZD-NN or 3DZD to obtain a probability (3DZD-NN) or a score (3DZD) that the protein pair belong to the same fold. In Table 3, we reported results obtained by using cutoff values of 0.6, 0.5, and 0.1 for 3DZD-NN mainchain, 3DZD-NN full-atom, and 3DZD, respectively, because these cutoffs gave the best F-scores among other cutoff values tried. In this table, cutoffs of 0.05 and 0.15 were used only for 3DZD to confirm that 0.1 is the best cutoff.

Supplementary Figure 1. AUC of ROC for fold classification performance

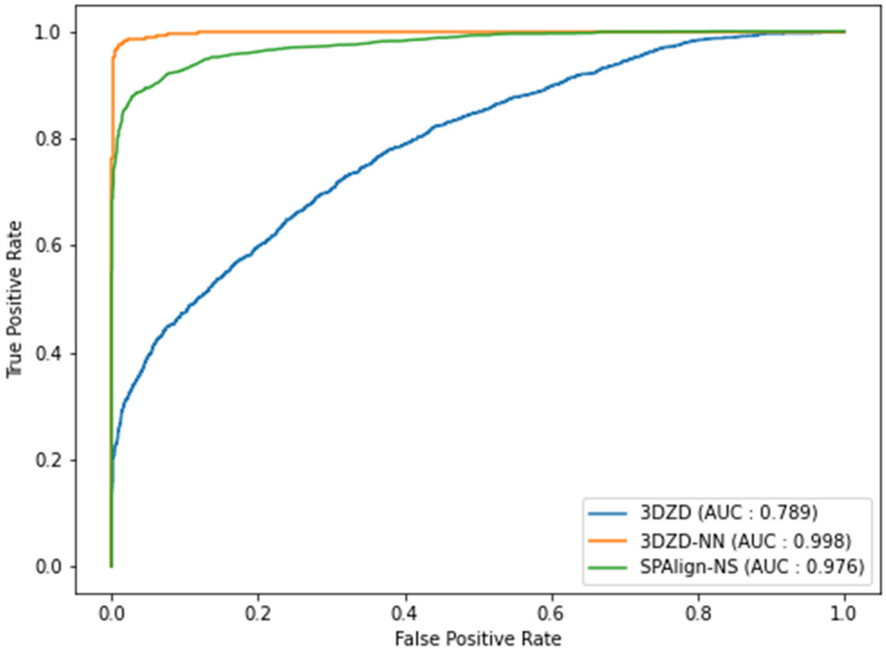

Fold recognition accuracy of 3DZD and 3DZD-NN were compared with SPalignNS. 5,000 protein pairs are randomly sampled with 2500 positive (i.e. the same fold pairs) and negative pairs, respectively. The pairs were ranked based on the scores of pairs computed by each method. The evaluation was made on 4,228 pairs where SPalignNS did not fail to run. For the rest of 772 cases, SPalignNS encountered an error and did not run.

Supplementary Figure 2. Database search results for examples shown in Fig. 4.

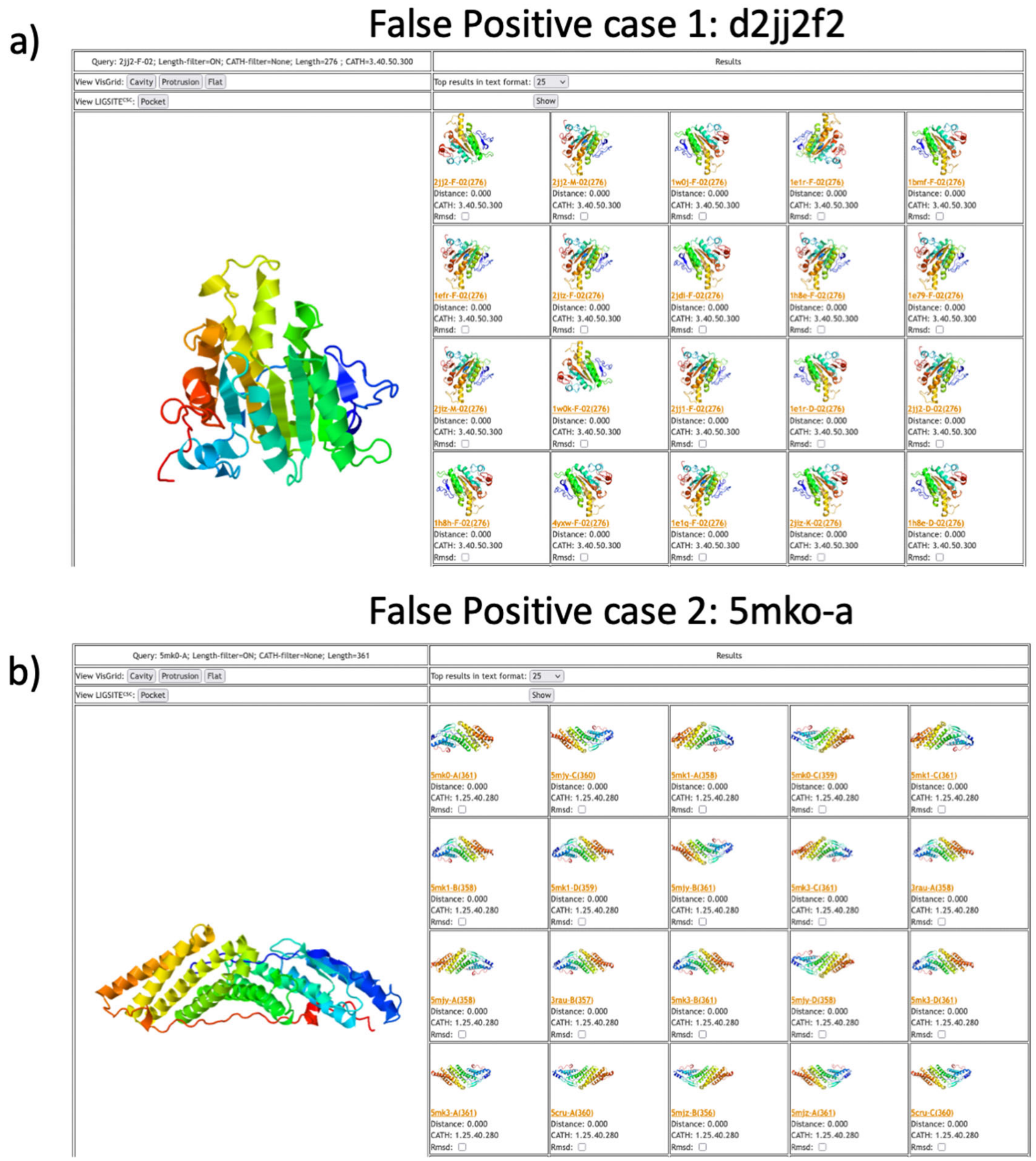

From two structures presented as examples of false positives in Fig. 4, d2jj2f2 and 5mko-a, the entire PDB was searched using 3DZD-NN with the main-chain representation. All structures retrieved within the top 25 for each query have the same fold as the query. Thus, although particular structure pairs with these queries were misrecognized as the same fold by 3DZD-NN and 3DZD as shown in Fig. 4, such relatively weak false positives do not affect the top hits in a database search.

Supplementary Table 2: Top-10 folds of AlphaFold2 models for individual species.

Methanocaldococcus jannaschii<br>1. TIM beta/alpha-barrel (c.1)<br>2. Non-globular all-alpha subunits of globular proteins (a.137)<br>3. L-aspartase-like (a.127)<br>4. Periplasmic binding protein-like II (c.94)<br>5. P-loop containing nucleoside triphosphate hydrolases (c.37)<br>6. ROP-like (a.30)<br>7. alpha-alpha superhelix (a.118)<br>8. PLP-dependent transferase-like (c.67)<br>9. Rhabdovirus nucleoprotein-like (a.260)<br>10. Bacillus chorismate mutase-like (d.79)	*Mycobacterium tuberculosis*<br>1. BAR/IMD domain-like (a.238)<br>2. ROP-like (a.30)<br>3. TIM beta/alpha-barrel (c.1)<br>4. Non-globular all-alpha subunits of globular proteins (a.137)<br>5. PLP-dependent transferase-like (c.67)<br>6. Intrinsically disordered proteins (g.88)<br>7. NAD(P)-binding Rossmann-fold domains (c.2)<br>8. L-aspartase-like (a.127)<br>9. Mediator hinge subcomplex-like (a.252)<br>10. alpha/beta-Hydrolases (c.69)
Staphylococcus aureus<br>1. ROP-like (a.30)<br>2. Non-globular all-alpha subunits of globular proteins (a.137)<br>3. TIM beta/alpha-barrel (c.1)<br>4. L-aspartase-like (a.127)<br>5. PLP-dependent transferase-like (c.67)<br>6. Acyl-CoA N-acyltransferases (Nat) (d.108)<br>7. S-adenosyl-L-methionine-depdt methyltransferases (c.66)<br>8. BAR/IMD domain-like (a.238)<br>9. Rhabdovirus nucleoprotein-like (a.260)<br>10. Periplasmic binding protein-like II (c.94)	*Escherichia coli*<br>1. TIM beta/alpha-barrel (c.1)<br>2. L-aspartase-like (a.127)<br>3. Periplasmic binding protein-like II (c.94)<br>4. Non-globular all-alpha subunits of globular proteins (a.137)<br>5. PLP-dependent transferase-like (c.67)<br>6. ROP-like (a.30)<br>7. Mediator hinge subcomplex-like (a.252)<br>8. Penicillin binding protein dimerisation domain (d.175)<br>9. IpaD-like (a.250)<br>10. Flavodoxin-like (c.23)
Saccharomyces cerevisiae<br>1. Non-globular all-alpha subunits of globular proteins (a.137)<br>2. ROP-like (a.30)<br>3. Mediator hinge subcomplex-like (a.252)<br>4. Intrinsically disordered proteins (g.88)<br>5. N-terminal domain of bifunctional PutA protein (a.176)<br>6. BAR/IMD domain-like (a.238)<br>7. L27 domain (a.194)<br>8. Histone-fold (a.22)<br>9. L-aspartase-like (a.127)<br>10. Spectrin repeat-like (a.7)	*Candida albicans*<br>1. Non-globular all-alpha subunits of globular proteins (a.137)<br>2. ROP-like (a.30)<br>3. Mediator hinge subcomplex-like (a.252)<br>4. N-terminal domain of bifunctional PutA protein (a.176)<br>5. Intrinsically disordered proteins (g.88)<br>6. BAR/IMD domain-like (a.238)<br>7. Spectrin repeat-like (a.7)<br>8. SRF-like (d.88)<br>9. L-aspartase-like (a.127)<br>10. Tetracyclin repressor-like, C-terminal domain (a.121)
Schizosaccharomyces pombe<br>1. Non-globular all-alpha subunits of globular proteins (a.137)<br>2. ROP-like (a.30)<br>3. Mediator hinge subcomplex-like (a.252)<br>4. N-terminal domain of bifunctional PutA protein (a.176)<br>5. BAR/IMD domain-like (a.238)<br>6. alpha-alpha superhelix (a.118)<br>7. Intrinsically disordered proteins (g.88)<br>8. Spectrin repeat-like (a.7)<br>9. L-aspartase-like (a.127)<br>10. SRF-like (d.88)	*Dictyostelium discoideum*<br>1. Non-globular all-alpha subunits of globular proteins (a.137)<br>2. ROP-like (a.30)<br>3. Mediator hinge subcomplex-like (a.252)<br>4. BAR/IMD domain-like (a.238)<br>5. Intrinsically disordered proteins (g.88)<br>6. N-terminal domain of bifunctional PutA protein (a.176)<br>7. CsrA-like (b.151)<br>8. L27 domain (a.194)<br>9. SRF-like (d.88)<br>10. Histone-fold (a.22)
Plasmodium falciparum<br>1. Non-globular all-alpha subunits of globular proteins (a.137)<br>2. ROP-like (a.30)<br>3. Mediator hinge subcomplex-like (a.252)<br>4. N-terminal domain of bifunctional PutA protein (a.176)<br>5. BAR/IMD domain-like (a.238)<br>6. L27 domain (a.194)<br>7. SRF-like (d.88)<br>8. Intrinsically disordered proteins (g.88)	*Trypanosoma cruzi*<br>1. Non-globular all-alpha subunits of globular proteins (a.137)<br>2. ROP-like (a.30)<br>3. Mediator hinge subcomplex-like (a.252)<br>4. N-terminal domain of bifunctional PutA protein (a.176)<br>5. Intrinsically disordered proteins (g.88)<br>6. L27 domain (a.194)<br>7. BAR/IMD domain-like (a.238)<br>8. AF2331-like (d.337)

9. Histone-fold (a.22)<br>10. Triple beta-spiral (b.83)	9. SRF-like (d.88)<br>10. CsrA-like (b.151)
Leishmania infantum<br>1. Non-globular all-alpha subunits of globular proteins (a.137)<br>2. ROP-like (a.30)<br>3. Mediator hinge subcomplex-like (a.252)<br>4. Intrinsically disordered proteins (g.88)<br>5. N-terminal domain of bifunctional PutA protein (a.176)<br>6. BAR/IMD domain-like (a.238)<br>7. L27 domain (a.194)<br>8. CsrA-like (b.151)<br>9. Spectrin repeat-like (a.7)<br>10. Histone-fold (a.22)	*Caenorhabditis elegans*<br>1. ROP-like (a.30)<br>2. Non-globular all-alpha subunits of globular proteins (a.137)<br>3. Mediator hinge subcomplex-like (a.252)<br>4. BAR/IMD domain-like (a.238)<br>5. Intrinsically disordered proteins (g.88)<br>6. Ferritin-like (a.25)<br>7. N-terminal domain of bifunctional PutA protein (a.176)<br>8. L-aspartase-like (a.127)<br>9. Spectrin repeat-like (a.7)<br>10. P-domain of calnexin/calreticulin (b.104)
Drosophila melanogaster<br>1. Non-globular all-alpha subunits of globular proteins (a.137)<br>2. ROP-like (a.30)<br>3. Intrinsically disordered proteins (g.88)<br>4. Mediator hinge subcomplex-like (a.252)<br>5. BAR/IMD domain-like (a.238)<br>6. N-terminal domain of bifunctional PutA protein (a.176)<br>7. alpha-alpha superhelix (a.118)<br>8. L27 domain (a.194)<br>9. L-aspartase-like (a.127)<br>10. Spectrin repeat-like (a.7)	*Danio rerio*<br>1. Non-globular all-alpha subunits of globular proteins (a.137)<br>2. ROP-like (a.30)<br>3. BAR/IMD domain-like (a.238)<br>4. Mediator hinge subcomplex-like (a.252)<br>5. Intrinsically disordered proteins (g.88)<br>6. N-terminal domain of bifunctional PutA protein (a.176)<br>7. Immunoglobulin-like beta-sandwich (b.1)<br>8. L27 domain (a.194)<br>9. P-domain of calnexin/calreticulin (b.104)<br>10. alpha-alpha superhelix (a.118)
Mus musculus<br>1. Non-globular all-alpha subunits of globular proteins (a.137)<br>2. ROP-like (a.30)<br>3. Immunoglobulin-like beta-sandwich (b.1)<br>4. BAR/IMD domain-like (a.238)<br>5. Mediator hinge subcomplex-like (a.252)<br>6. Intrinsically disordered proteins (g.88)<br>7. alpha-alpha superhelix (a.118)<br>8. Tex N-terminal region-like (a.294)<br>9. L27 domain (a.194)<br>10. N-terminal domain of bifunctional PutA protein (a.176)	*Rattus norvegicus*<br>1. Non-globular all-alpha subunits of globular proteins (a.137)<br>2. ROP-like (a.30)<br>3. BAR/IMD domain-like (a.238)<br>4. Mediator hinge subcomplex-like (a.252)<br>5. Immunoglobulin-like beta-sandwich (b.1)<br>6. Intrinsically disordered proteins (g.88)<br>7. Tex N-terminal region-like (a.294)<br>8. alpha-alpha superhelix (a.118)<br>9. L27 domain (a.194)<br>10. N-terminal domain of bifunctional PutA protein (a.176)
Homo sapiens<br>1. Non-globular all-alpha subunits of globular proteins (a.137)<br>2. ROP-like (a.30)<br>3. BAR/IMD domain-like (a.238)<br>4. Immunoglobulin-like beta-sandwich (b.1)<br>5. Mediator hinge subcomplex-like (a.252)<br>6. Intrinsically disordered proteins (g.88)<br>7. N-terminal domain of bifunctional PutA protein (a.176)<br>8. Pyruvate kinase C-terminal domain-like (c.49)<br>9. Interferon-induced guanylate-binding protein 1 (GBP1), C-terminal domain (a.114)<br>10. L27 domain (a.194)	*Oryza sativa*<br>1. Non-globular all-alpha subunits of globular proteins (a.137)<br>2. ROP-like (a.30)<br>3. Mediator hinge subcomplex-like (a.252)<br>4. Leu-rich repeat, LRR (right-handed beta-alpha superhelix) (c.10)<br>5. SRF-like (d.88)<br>6. CsrA-like (b.151)<br>7. YefM-like (d.306)<br>8. alpha-alpha superhelix (a.118)<br>9. Histone-fold (a.22)<br>10. L27 domain (a.194)
Zea mays<br>1. Non-globular all-alpha subunits of globular proteins (a.137)<br>2. ROP-like (a.30)<br>3. Mediator hinge subcomplex-like (a.252)<br>4. YheA-like (a.281)<br>5. SRF-like (d.88)<br>6. CsrA-like (b.151)<br>7. L27 domain (a.194)<br>8. alpha-alpha superhelix (a.118)	*Arabidopsis thaliana*<br>1. Non-globular all-alpha subunits of globular proteins (a.137)<br>2. ROP-like (a.30)<br>3. Mediator hinge subcomplex-like (a.252)<br>4. alpha-alpha superhelix (a.118)<br>5. Leu-rich repeat, LRR (right-handed beta-alpha superhelix) (c.10)<br>6. SRF-like (d.88)<br>7. CsrA-like (b.151)<br>8. Histone-fold (a.22)

9. N-terminal domain of bifunctional PutA protein (a.176)<br>10. triple barrel (b.65)	9. N-terminal domain of bifunctional PutA protein (a.176)<br>10. Spectrin repeat-like (a.7)
Glycine max<br>1. Non-globular all-alpha subunits of globular proteins (a.137)<br>2. ROP-like (a.30)<br>3. Mediator hinge subcomplex-like (a.252)<br>4. Leu-rich repeat, LRR (right-handed β–α superhelix) (c.10)<br>5. alpha-alpha superhelix (a.118)<br>6. SRF-like (d.88)<br>7. Spectrin repeat-like (a.7)<br>8. CsrA-like (b.151)<br>9. L27 domain (a.194)<br>10. N-terminal domain of bifunctional PutA protein (a.176)	

Commonly appeared folds with the SUPERFAMILY2.0 statistics shown in Supplementary Table 4 are underlined. Folds in α-class has a SCOP ID stating from a., an ID of a β-class fold starts from b., α/β and α+β class folds have ID with c. and d. respectively, folds with g. are small proteins.

Supplementary Table 3. Statistics of SUPERFAMILY 2.0 of the 21 species.

**Species**	**SUPERFAMILY ID**	**# Sequences**	**# sequence with assignment**	**# domains with assignment**
Arabidopsis thaliana	at	35,381	22,134	32,197
Caenorhabditis elegans	cl	30,250	16,476	29,666
Candida albicans	al	6,165	3,795	5,360
Danio rerio	da	43,153	31,400	67,350
Dictyostelium discoideum	dt	13,263	6,955	11,119
Drosophila melanogaster	dd	26,950	17,699	38,800
Escherichia coli	ec	4,141	3,010	4,434
Glycine max	yg	55,787	36,697	51,734
Homo sapiens	hs	99,458	60,672	115,355
Leishmania infantum	lh	8,216	4,294	6,092
Methanocaldococcus jannaschii	jM	1,771	1,177	1,614
Mus musculus	mm	52,998	34,563	68,456
Mycobacterium tuberculosis	7EO	3,994	2,814	3,984
Oryza sativa	AnK	66,338	32,867	51,632
Plasmodium falciparum	pl	5,385	2735	4,219
Rattus norvegicus	rn	25,724	18,941	36,627
Saccharomyces cerevisiae	xs	6,692	3,654	5,383
Schizosaccharomyces pombe	po	5,035	3,356	5,024
Staphylococcus aureus	brP	1,981	1,512	2,375
Trypanosoma cruzi	uz	10,320	4,719	6,449
Zea mays	e0I	63,540	34,145	45,617

The statistics from the SUPERFAMILY 2.0 database for the same 21 species in Table 1. The annotations were downloaded on December 10, 2021. The SCOP superfamily assigned for each domain by SUPERFAMILY was counted for individual species in the SCOP fold level.

Supplementary Table 4. Top-10 Folds in SUPERFAMILY 2.0 for individual species.

***Methanocaldococcus jannaschii* (jM)**<br>1. P-loop cont. nucleoside triphosphate hydrolases (c.37)<br>2. Ferredoxin-like (d.58)<br>3. TIM beta/alpha-barrel (c.1)<br>4. DNA/RNA-binding 3-helical bundle (a.4)<br>5. S-a.-L-methionine-dept methyltransferases (c.66)<br>6. Adenine nucleotide alpha hydrolase-like (c.26)<br>7. OB-fold (b.40)<br>8. NAD(P)-binding Rossmann-fold domains (c.2)<br>9. Flavodoxin-like (c.23) a<br>10. Homing endonuclease-like (d.95)	***Mycobacterium tuberculosis* (7EO)**<br>1. P-loop containing nucleoside triphosphate hydrolases (c.37)<br>2. Ferritin-like (a.25)<br>3. DNA/RNA-binding 3-helical bundle (a.4)<br>4. NAD(P)-binding Rossmann-fold domains (c.2)<br>5. TIM beta/alpha-barrel (c.1)<br>6. Ferredoxin-like (d.58)<br>7. alpha/beta-Hydrolases (c.69)<br>8. S-a.-L-methionine-dependent methyltransferases (c.66)<br>9. FAD/NAD(P)-binding domain (c.3)<br>10. Thiolase-like (c.95)
***Staphylococcus aureus* (brP)**<br>1. P-loop cont. nucleoside triphosphate hydrolases (c.37)<br>2. immunoglobulin/albumin-binding domain-like (a.8)<br>3. TIM beta/alpha-barrel (c.1)<br>4. DNA/RNA-binding 3-helical bundle (a.4)<br>5. NAD(P)-binding Rossmann-fold domains (c.2)<br>6. Ferredoxin-like (d.58)<br>7. OB-fold (b.40)<br>8. Flavodoxin-like (c.23)<br>9. MFS general substrate transporter (f.38)<br>10. FAD/NAD(P)-binding domain (c.3)	***Escherichia coli* (ec)**<br>1. P-loop cont. nucleoside triphosphate hydrolases (c.37)<br>2. DNA/RNA-binding 3-helical bundle (a.4)<br>3. TIM beta/alpha-barrel (c.1)<br>4. Ferredoxin-like (d.58)<br>5. NAD(P)-binding Rossmann-fold domains (c.2)<br>6. Ribonuclease H-like motif (c.55)<br>7. Flavodoxin-like (c.23)<br>8. Periplasmic binding protein-like II (c.94)<br>9. MFS general substrate transporter (f.38)<br>10. OB-fold (b.40)
***Saccharomyces cerevisiae* (xs)**<br>1. P-loop cont. nucleoside triphosphate hydrolases (c.37)<br>2. alpha-alpha superhelix (a.118)<br>3. 7-bladed beta-propeller (b.69)<br>4. Ferredoxin-like (d.58)<br>5. Ribonuclease H-like motif (c.55)<br>6. Protein kinase-like (PK-like) (d.144)<br>7. TIM beta/alpha-barrel (c.1)<br>8. DNA/RNA-binding 3-helical bundle (a.4)<br>9. NAD(P)-binding Rossmann-fold domains (c.2)<br>10. MFS general substrate transporter (f.38)	***Candida albicans* (al)**<br>1. P-loop containing nucleoside triphosphate hydrolases (c.37)<br>2. alpha-alpha superhelix (a.118)<br>3. Ferredoxin-like (d.58)<br>4. 7-bladed beta-propeller (b.69)<br>5. TIM beta/alpha-barrel (c.1)<br>6. NAD(P)-binding Rossmann-fold domains (c.2)<br>7. Protein kinase-like (PK-like) (d.144)<br>8. MFS general substrate transporter (f.38)<br>9. Ribonuclease H-like motif (c.55)<br>10. DNA/RNA-binding 3-helical bundle (a.4)
***Schizosaccharomyces pombe* (po)**<br>1. P-loop cont. nucleoside triphosphate hydrolases (c.37)<br>2. alpha-alpha superhelix (a.118)<br>3. 7-bladed beta-propeller (b.69)<br>4. Ferredoxin-like (d.58)<br>5. Protein kinase-like (PK-like) (d.144)<br>6. TIM beta/alpha-barrel (c.1)<br>7. DNA/RNA-binding 3-helical bundle (a.4)<br>8. Ribonuclease H-like motif (c.55)<br>9. NAD(P)-binding Rossmann-fold domains (c.2)<br>10. OB-fold (b.40)	***Dictyostelium discoideum* (dt)**<br>1. P-loop containing nucleoside triphosphate hydrolases (c.37)<br>2. alpha-alpha superhelix (a.118)<br>3. Protein kinase-like (PK-like) (d.144)<br>4. Ferredoxin-like (d.58)<br>5. 7-bladed beta-propeller (b.69)<br>6. Immunoglobulin-like beta-sandwich (b.1)<br>7. Leu-rich repeat, LRR (right-handed $\beta-\alpha$ superhelix) (c.10)<br>8. NAD(P)-binding Rossmann-fold domains (c.2)<br>9. Ribonuclease H-like motif (c.55)<br>10. RING/U-box (g.44)
***Plasmodium falciparum* (pl)**<br>1. P-loop cont. nucleoside triphosphate hydrolases (c.37)<br>2. Duffy binding domain-like (a.264)<br>3. alpha-alpha superhelix (a.118)<br>4. Ferredoxin-like (d.58)<br>5. 7-bladed beta-propeller (b.69)<br>6. Protein kinase-like (PK-like) (d.144)<br>7. OB-fold (b.40)	***Trypanosoma cruzi* (uz)**<br>1. P-loop cont. nucleoside triphosphate hydrolases (c.37)<br>2. 6-bladed beta-propeller (b.68)<br>3. Concanavalin A-like lectins/glucanases (b.29)<br>4. alpha-alpha superhelix (a.118)<br>5. Protein kinase-like (PK-like) (d.144)<br>6. 7-bladed beta-propeller (b.69)<br>7. Ferredoxin-like (d.58)

8. EF Hand-like (a.39)<br>9. Ribonuclease H-like motif (c.55)<br>10. Long alpha-hairpin (a.2)	8. Zincin-like (d.92)<br>9. Single-stranded right-handed beta-helix (b.80)<br>10. Leu-rich repeat, LRR (right-handed $\beta-\alpha$ superhelix) (c.10)
***Leishmania infantum* (lh)**<br>1. P-loop cont. nucleoside triphosphate hydrolases (c.37)<br>2. alpha-alpha superhelix (a.118)<br>3. Protein kinase-like (PK-like) (d.144)<br>4. 7-bladed beta-propeller (b.69)<br>5. Ferredoxin-like (d.58)<br>6. Leu-rich repeat, LRR (r-handed $\beta-\alpha$ superhelix) (c.10)<br>7. Ribonuclease H-like motif (c.55)<br>8. Long alpha-hairpin (a.2)<br>9. S-a.-L-methionine-dpdt methyltransferases (c.66)<br>10. TIM beta/alpha-barrel (c.1)	***Caenorhabditis elegans* (cl)**<br>1. Immunoglobulin-like beta-sandwich (b.1)<br>2. Class A G protein-coupled receptor (GPCR)-like (f.13)<br>3. P-loop containing nucleoside triphosphate hydrolases (c.37)<br>4. Protein kinase-like (PK-like) (d.144)<br>5. alpha-alpha superhelix (a.118)<br>6. Ferredoxin-like (d.58)<br>7. Knottins (small inhibitors, toxins, lectins) (g.3)<br>8. Glucocorticoid receptor-like (DNA-binding domain) (g.39)<br>9. BPTI-like (g.8)<br>10. DNA/RNA-binding 3-helical bundle (a.4)
***Drosophila melanogaster* (dd)**<br>1. Immunoglobulin-like beta-sandwich (b.1)<br>2. beta-beta-alpha zinc fingers (g.37)<br>3. Knottins (small inhibitors, toxins, lectins) (g.3)<br>4. Spectrin repeat-like (a.7)<br>5. P-loop cont. nucleoside triphosphate hydrolases (c.37)<br>6. Ferredoxin-like (d.58)<br>7. alpha-alpha superhelix (a.118)<br>8. Protein kinase-like (PK-like) (d.144)<br>9. Glucocorticoid receptor-like (DNA-bdg domain) (g.39)<br>10. SH3-like barrel (b.34)	***Danio rerio* (da)**<br>1. beta-beta-alpha zinc fingers (g.37)<br>2. Immunoglobulin-like beta-sandwich (b.1)<br>3. P-loop containing nucleoside triphosphate hydrolases (c.37)<br>4. Knottins (small inhibitors, toxins, lectins) (g.3)<br>5. Protein kinase-like (PK-like) (d.144)<br>6. alpha-alpha superhelix (a.118)<br>7. Leu-rich repeat, LRR (right-handed $\beta-\alpha$ superhelix) (c.10)<br>8. Concanavalin A-like lectins/glucanases (b.29)<br>9. DNA/RNA-binding 3-helical bundle (a.4)<br>10. SH3-like barrel (b.34)
***Mus musculus* (mm)**<br>1. Immunoglobulin-like beta-sandwich (b.1)<br>2. beta-beta-alpha zinc fingers (g.37)<br>3. P-loop cont. nucleoside triphosphate hydrolases (c.37)<br>4. Class A G protein-coupled receptor (GPCR)-like (f.13)<br>5. alpha-alpha superhelix (a.118)<br>6. Knottins (small inhibitors, toxins, lectins) (g.3)<br>7. Ferredoxin-like (d.58)<br>8. DNA/RNA-binding 3-helical bundle (a.4)<br>9. Protein kinase-like (PK-like) (d.144)<br>10. PH domain-like barrel (b.55)	***Rattus norvegicus* (rn)**<br>1. Immunoglobulin-like beta-sandwich (b.1)<br>2. beta-beta-alpha zinc fingers (g.37)<br>3. Class A G protein-coupled receptor (GPCR)-like (f.13)<br>4. P-loop containing nucleoside triphosphate hydrolases (c.37)<br>5. alpha-alpha superhelix (a.118)<br>6. Knottins (small inhibitors, toxins, lectins) (g.3)<br>7. Ferredoxin-like (d.58)<br>8. DNA/RNA-binding 3-helical bundle (a.4)<br>9. SH3-like barrel (b.34)<br>10. Protein kinase-like (PK-like) (d.144)
***Homo sapiens* (hs)**<br>1. Immunoglobulin-like beta-sandwich (b.1)<br>2. beta-beta-alpha zinc fingers (g.37)<br>3. P-loop cont. nucleoside triphosphate hydrolases (c.37)<br>4. alpha-alpha superhelix (a.118)<br>5. Knottins (small inhibitors, toxins, lectins) (g.3)<br>6. Ferredoxin-like (d.58)<br>7. Protein kinase-like (PK-like) (d.144)<br>8. SH3-like barrel (b.34)<br>9. DNA/RNA-binding 3-helical bundle (a.4)<br>10. PH domain-like barrel (b.55)	***Oryza sativa* (AnK)**<br>1. Ribonuclease H-like motif (c.55)<br>2. DNA/RNA polymerases (e.8)<br>3. Retrovirus zinc finger-like domains (g.40)<br>4. Leu-rich repeat, LRR (r.-handed $\beta-\alpha$ superhelix) (c.10)<br>5. P-loop containing nucleoside triphosphate hydrolases (c.37)<br>6. Protein kinase-like (PK-like) (d.144)<br>7. Acid proteases (b.50)<br>8. alpha-alpha superhelix (a.118)<br>9. Ferredoxin-like (d.58)<br>10. Cysteine proteinases (d.3)
***Zea mays* (e0I)**<br>1. Protein kinase-like (PK-like) (d.144)<br>2. P-loop cont. nucleoside triphosphate hydrolases (c.37)<br>3. alpha-alpha superhelix (a.118)<br>4. Ferredoxin-like (d.58)<br>5. DNA/RNA-binding 3-helical bundle (a.4)<br>6. TIM beta/alpha-barrel (c.1)	***Arabidopsis thaliana* (at)**<br>1. P-loop containing nucleoside triphosphate hydrolases (c.37)<br>2. Protein kinase-like (PK-like) (d.144)<br>3. alpha-alpha superhelix (a.118)<br>4. Leu-rich repeat, LRR (r.-handed $\beta-\alpha$ superhelix) (c.10)<br>5. Ferredoxin-like (d.58)<br>6. DNA/RNA-binding 3-helical bundle (a.4)

7. RING/U-box (g.44)<br>8. Leu-rich repeat, LRR (r-handed β–α superhelix) (c.10)<br>9. NAD(P)-binding Rossmann-fold domains (c.2)<br>10. 7-bladed beta-propeller (b.69)	7. RING/U-box (g.44)<br>8. F-box domain (a.158)<br>9. Cysteine-rich domain (g.49)<br>10. TIM beta/alpha-barrel (c.1)
***Glycine max* (yg)**<br>1. P-loop cont. nucleoside triphosphate hydrolases (c.37)<br>2. Protein kinase-like (PK-like) (d.144)<br><u>3. Leu-rich repeat, LRR (r.-handed β–α superhelix) (c.10)</u><br><u>4. alpha-alpha superhelix (a.118)</u><br>5. Ferredoxin-like (d.58)<br>6. DNA/RNA-binding 3-helical bundle (a.4)<br>7. RING/U-box (g.44)<br>8. TIM beta/alpha-barrel (c.1)<br>9. NAD(P)-binding Rossmann-fold domains (c.2)<br>10. 7-bladed beta-propeller (b.69)	

The Genome ID used in SUPERFAMILY is noted after each species name. Folds commonly appeared in the Alphafold2 models in Supplementary Table 2 and in this SUPERFAMILY database are underlined.

Supplementary Table 5. The top 10 most abundant folds by Gerstein (1998)

Escherichia coli **(EC)**	*Saccharomyces cerevisiae* **(SC)**
1. NAD(P)-binding Rossmann Fold (c.18)	1. P-loop Containing NTP Hydrolases (c.24)
2. Flavodoxin-like (c.13)	2. Ribonuclease H-like motif (c.38)
3. TIM-Barrel (c.1)	3. NAD(P)-binding Rossmann Fold (c.18)
4. Like Ferrodoxin (d.31)	4. TIM-Barrel (c.1)
5. Ribonuclease H-like motif (c.38)	5. Like Ferrodoxin (d.31)
6. P-loop Containing NTP Hydrolases (c.24)	6. Long Alpha-hairpin (a.2)
7. Thiamin-binding (c.23)	7. Thiamin-binding (c.23)
8. FAD/NAD(P)-binding (c.4)	8. GroES-like (b.21)
9. GroES-like (b.21)	9. Thioredoxin-like (c.30)
10. OB-fold (b.24)	10. FAD/NAD(P)-binding (c.4)
Methanocaldococcus jannaschii **(MJ)**	
1. Like Ferrodoxin (d.31)	
2. P-loop containing NTP Hydrolases (c.24)	
3. TIM-Barrel (c.1)	
4. FAD/NAD(P)-binding (c.4)	
5. Thiamin-binding (c.23)	
6. NAD(P)-binding Rossman Fold (c.18)	
7. ATP Pyrophosphatases (c.15)	
8. Flavodoxin-like (c.13)	
9. Reductase/Elongation Factor Domain (b.27)	
10. Asp-carbomoyltransferase, Cat.-chain (c.56)	

The top 10 most abundant folds in the three species taken from Fig. 1. in the paper by M. Gerstein, Patterns of protein-fold usage in eight microbial genomes: a comprehensive structural census. *Proteins* **33**, 518-534, (1998). Among eight species analyzed in their work, three species that are common with Supplementary Table 2 are listed. Folds with underline are those which are in common with Supplementary Table 2. In the parentheses, SCOP codes are shown.

Supplementary Table 6. The top 5 most abundant folds by Kihara & Skolnick (2004).

Escherichia coli	*Saccharomyces cerevisiae*
1. Rossmann fold (3.40.50)	1. Rossmann fold (3.40.50)
2. Alpha-Betta plaits (3.30.70)	2. Alpha-Betta plaits (3.30.70)
3. TIM barrel (3.20.20)	3. Immunoglobin-like (2.60.40)
4. Arc repressor mutant subunit A (1.10.10)	4. Arc repressor mutatant subunit A (1.10.10)
5. Immunoglobin-like (2.60.40)	5. Kinase (3.30.200)

The top 5 most abundant folds in the two species from Table IIIB in the paper by D. Kihara & J. Skolnick, Microbial genomes have over 72% structure assignment by the threading algorithm PROSPECTOR_Q, *Proteins,* **55**, 464-473, 2004. Only top 5 folds are listed here because their work only showed top 5. Among five species analyzed in their work, these two species were in common with Supplementary Table 2. Folds with underline are commonly appeared in Supplementary Table 2. In the parentheses, CATH codes are shown.
